# Mechanistic exploration of obesity-related indicators and motor cognitive risk syndrome: a mediated effect based on C-reactive protein triglyceride glucose index

**DOI:** 10.3389/fnagi.2025.1623148

**Published:** 2025-07-30

**Authors:** Zhongqiang Guo, Shuang Deng, Li Li, Min Liu

**Affiliations:** ^1^School of Nursing and Health, Henan University, Kaifeng, Henan, China; ^2^Tianhua College, Shanghai Normal University, Shanghai, China

**Keywords:** obesity indicators, motor cognitive risk syndrome, metabolic disorders, mediation effect analysis, threshold effect analysis, multiple logistic regression analysis

## Abstract

**Objective:**

To investigate the relationships between obesity-related indicators and motor cognitive risk syndrome (MCR), as well as the mediating role of the C-reactive protein triglyceride glucose index (CTI).

**Methods:**

The aim of this study was to provide evidence-based support to optimize MCR screening programs and develop prevention strategies for cognitive impairment in elderly individuals. This study utilized data from the China Health and Retirement Longitudinal Study (CHARLS) in 2015, and 5,665 participants were ultimately included. The independent variables were obesity-related indicators (WC, Waist circumference; WHtR, waist to height ratio; BRI, body roundness index; BMI, body mass index), and the mediating variable was CTI. Multiple logistic regression analysis, threshold effect analysis, and curve fitting analysis were used to analyze the relationships between obesity-related indicators and MCR. The mediation analysis method was used to observe the mediating effect of CTI.

**Results:**

BMI, WC, WHtR, and BRI all significantly increased the risk of MCR. Threshold analysis revealed a nonlinear BRI-MCR association (inflection point = 2.45, *P* = 0.041), whereas the association with WHtR was linear (*P* = 0.069). Mediation analysis revealed that the CTI mediated 20.99% of the effect of the WHtR on MCR, 25.55% of the effect of WC, and 21.74% of the effect of the BRI. The overall effect, direct effect, and indirect effect are all significant.

**Conclusion:**

This study, which is based on CHARLS data, revealed that obesity-related indicators (WC, WHtR, BRI) significantly correlate with MCR risk. Metabolic disorders mediate the association of WHtR with MCR, confirming their central role in the link between obesity and cognition. Threshold effects were observed. The proposed method is to incorporate WHtR and CTI into community health assessments for early cognitive impairment screening, offering evidence for targeted interventions in resource-limited settings.

## 1 Introduction

According to the 2023 World Alzheimer’s Report, the number of patients with dementia worldwide is projected to surge from 57.4 million in 2019 to 152.8 million by 2050, with low- and middle-income countries (LMICs) accounting for 68% of this increase (Shaffi et al., 2024). This growth is attributed primarily to the acceleration of population aging, particularly in emerging nations. For example, by 2050, the number of patients with dementia in Asia and Africa is expected to reach 67.2 million and 46.8 million, respectively, far surpassing Europe (22.9 million) and the Americas (58 million) (Stevenson et al., 2024). Although high-income countries are experiencing slower growth, the total number of patients will still significantly increase. For example, the number of dementia patients in the United States, Canada, and China is anticipated to increase by 51, 59, and 70%, respectively, by 2030 (Choi et al., 2024). This trend presents a serious challenge to the global healthcare system, particularly for LMICs with limited resources, which must contend with the dual pressures of surging care demand and inadequate medical resources.

Motor cognitive risk syndrome (MCR) is characterized by subjective cognitive decline and objective gait delay, and it is a strong predictor of dementia transformation. A cross-national cohort study revealed that the risk of developing dementia in patients with MCR was 1.9 times greater than that in cognitively normal individuals (HR = 1.9, 95% CI: 1.5–2.3), whereas another study reported a 3.27-fold increase in risk (HR = 3.27, 95% CI: 1.55–6.90), which was particularly significantly associated with vascular dementia (Verghese et al., 2013). However, its pathological mechanism has not been fully elucidated, especially the pathways through which metabolic disorders contribute to the occurrence and development of MCR.

Obesity-related metabolic disorders may be a potential regulatory pathway for the onset of MCR. Prospective studies have shown that central obesity indicators, such as WHtR, can lead to cognitive decline through the neuroinflammatory cascade and insulin resistance. The C-reactive protein triglyceride glucose index (CTI), a novel composite biomarker that integrates inflammation and lipid metabolism, has shown significant potential in predicting neurodegenerative diseases (Biswas et al., 2021; Cai et al., 2013; He et al., 2016). However, there are still limitations in current research that are reflected in the lack of systematic comparisons of the predictive efficacy of different anthropometric indicators for MCR, the lack of validation of the mediating role of lipid metabolism, and the lack of quantification of the potential nonlinear dose–response relationship between obesity parameters and MCR risk.

This study is based on the China Health and Retirement Longitudinal Study (CHARLS) to explore the relationships among obesity-related indicators. Logistic regression models were used to calculate odds ratios (ORs) and 95% confidence intervals (CIs) to evaluate the associations between obesity and lipid metabolism indicators (continuous and quartiles) and MCR. These models were initially adjusted for age, sex, and education level and then further adjusted for BMI, smoking status, alcohol consumption, and chronic diseases. A stratified analysis was conducted on the basis of sex, education level, smoking status, and alcohol consumption to examine the associations between obesity and lipid metabolism indicators and MCR. After adjusting for potential confounding factors, the relationships among obesity-related indicators, lipid metabolism indicators, and MCR were explored through smooth curve fitting and threshold effects. A two-tailed *p*-value of ≤ 0.05 was considered statistically significant.

## 2 Materials and methods

### 2.1 Study population

The research subjects for this study are drawn from the China Health and Retirement Longitudinal Study (CHARLS), a comprehensive longitudinal study covering the whole country and targeting the population aged 45 and above. The research design and evaluation methods of CHARLS have been extensively described in previous studies (Ding et al., 2020; Liu et al., 2022). In brief, through a multistage probability sampling method, eligible individuals were recruited from 450 communities and administrative villages across 28 provinces in China (Bo and Yating, 2023; Liao et al., 2021). After enrollment, participants completed a standardized questionnaire and relevant physical examinations. Afterward, follow-up was conducted every two years. The CHARLS project has been approved by the Biomedical Ethics Committee of Peking University (IRB00001052-1015), and all participants have signed informed consent forms, agreeing to data sharing and disclosure (Hua et al., 2021; Wan et al., 2021).

**FIGURE 1:**
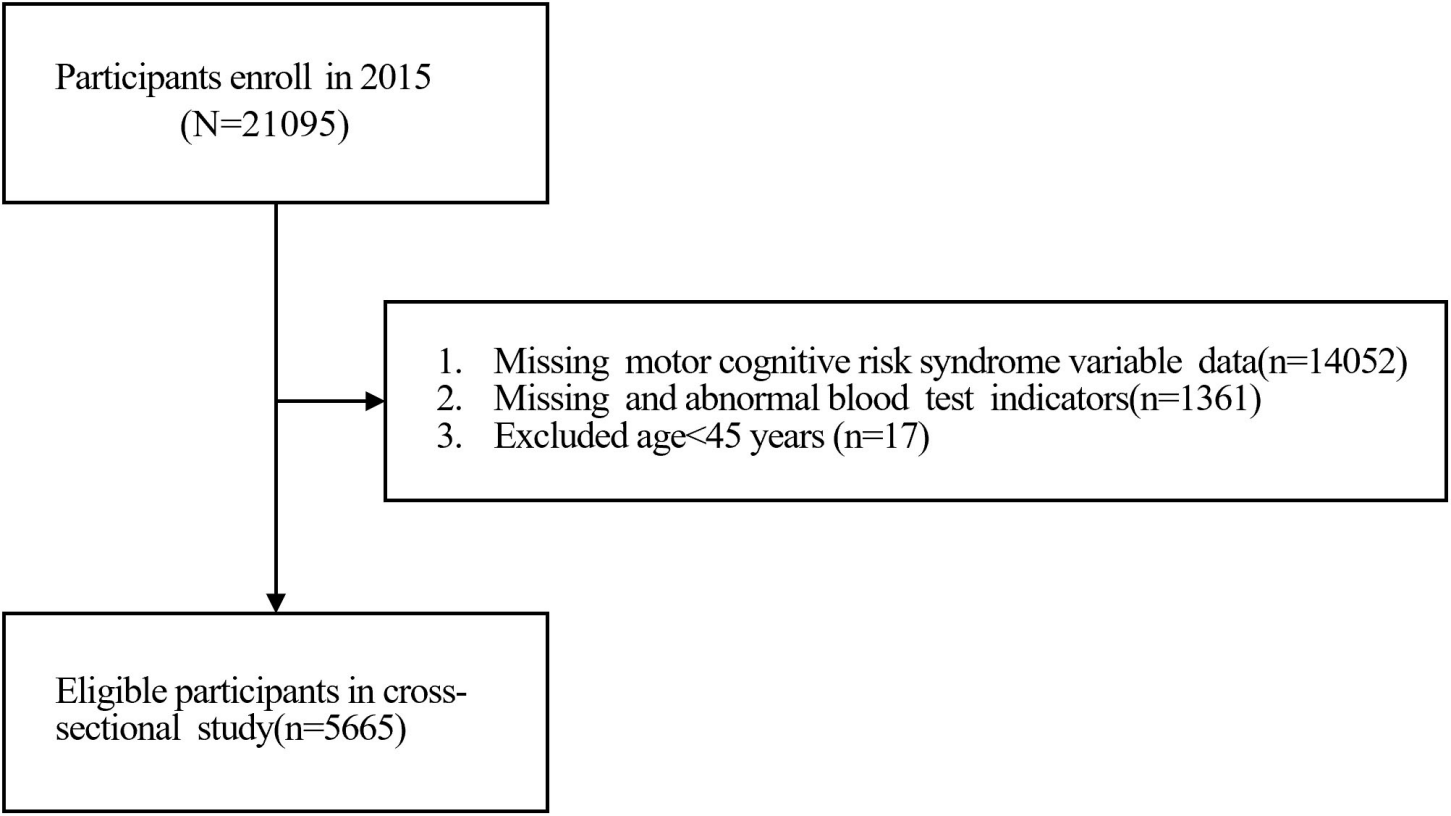
Flow diagram for participants included in the study. MCR, motoric cognitive risk syndrome.

This study focuses on data collected by CHARLS in 2015. The initial recruitment sample size was 21,095 people. During the data screening process, 14,052 participants with missing dependent variable data, 1,361 participants with missing and abnormal blood test indicators, and 17 participants under the age of 45 years were excluded. After the above exclusion criteria were met, 5,665 participants were ultimately included in the subsequent analysis (Figure 1 and Supplementary Table 1).

### 2.2 Evaluation of indicators related to obesity and lipid metabolism

#### 2.2.1 Indicators

Body mass index (BMI): BMI was calculated by dividing weight (in kilograms) by the square of height (in meters). Height and weight were measured using a height and weight scale, during which participants wore lightweight clothing and no shoes (Kali et al., 2022; Merrigan et al., 2022; Tornero-Aguilera et al., 2022).


$$\text{BMI}=\frac{W\,e\,i\,g\,h\,t\,\left(k\,g\right)}{H\,e\,i\,g\,h\,t\,{\left(m\right)}^{2}}$$

Waist circumference (WC): WC is measured with a tape measure at the midpoint between the lower edge of the rib and the upper edge of the iliac crest (Takahara et al., 2012).

Waist-to-height ratio (WHtR): This ratio is calculated by dividing waist circumference by height. The WHtR is an indicator of central obesity (Qin et al., 2023).


$$\text{WHtR}=\frac{W\,a\,i\,s\,t\,C\,i\,r\,c\,u\,m\,f\,e\,r\,e\,n\,c\,e\,\left(m\right)}{H\,e\,i\,g\,h\,t\,\left(m\right)}$$

Obesity index (BRI): Based on the ratio of waist circumference to height, the aim is to reflect an individual’s fat distribution through simplified body shape features. The BRI is more sensitive than the BMI in reflecting abdominal fat accumulation and has greater predictive power for assessing central obesity (Chen et al., 2021; Xu et al., 2021).


$$\text{BRI}=364.2-365.5\,\sqrt{1-\left(\frac{{\left(W\,C\,\left(m\right)/\left(2\,\pi \right)\right)}^{2}}{{\left(0.5\times H\,e\,i\,g\,h\,t\,\left(m\right)\right)}^{2}}\right)}$$

The ABSI is derived from waist circumference (WC), height, and body mass index (BMI) using the following formula (Yang et al., 2024).


$$\text{ABSI}=\frac{W\,a\,i\,s\,t\,C\,i\,r\,c\,u\,m\,f\,e\,r\,e\,n\,c\,e\,\left(m\right)}{{\left[H\,e\,i\,g\,h\,t\,{\left(m\right)}^{1/2}\times B\,M\,I\,\left(\mathrm{kg}/m^{2}\right)\right]}^{2/3}}$$

The CI is computed using waist circumference (WC), weight, and height with the following formula (Sabbah et al., 2012).


$$\text{CI}=\frac{W\,a\,i\,s\,t\,C\,i\,r\,c\,u\,m\,f\,e\,r\,e\,n\,c\,e\,\left(m\right)}{0.109\times \sqrt{\frac{W\,e\,i\,g\,h\,t\,\left(\mathrm{kg}\right)}{H\,e\,i\,g\,h\,t\,\left(m\right)}}}$$

#### 2.2.2 Lipid metabolism indicators

C-reactive protein triglyceride glucose index (CTI): The formula for detecting C-reactive protein, triglyceride, and glucose levels in blood samples is as follows:

CTI=0.412×ln(C-reactiveprotein[CRP])+ln(triglycerides [mg/dL] × fasting blood glucose [mg/dL]/2) (Ren et al., 2025). As a novel composite biomarker that integrates inflammation and lipid metabolism, it has shown significant potential in predicting neurodegenerative diseases (Li et al., 2024).

#### 2.2.3 Assessment of MCR

The characteristics of MCR are subjective cognitive decline, slow walking speed, normal mobility, and no dementia. Cognitive function is evaluated by asking participants to self-rate their current memory. Individuals who rate their memory as average or poor are considered to have cognitive problems, whereas others are considered to have no cognitive problems. Slow walking speed is determined to be one standard deviation lower than the average walking speed with respect to age- and sex-specific populations at baseline. In this study, the cutoff values used for classifying slow gait speed are as follows: males < 75 years = 0.44 m/s, males ≥ 75 years = 0.35 m/s, females < 75 years = 0.41 m/s, females ≥ 75 years = 0.33 m/s (Xu et al., 2025).

#### 2.2.4 Evaluation of potential confounding factors

Age, sex, marital status, place of residence, education level, smoking status, and alcohol consumption information were obtained through a unified structured questionnaire. According to the literature published previously (Huang et al., 2013; Smith et al., 2012), studies using the CHARLS divide education into four categories: illiterate, partially primary school/literate or able to write, completed primary school, and junior high school or above. Smoking status was divided into no and yes. The frequency of drinking is defined as follows (Chen H. et al., 2024). Current nondrinkers refer to those who never or almost never drink, and the option “none” is included. Occasional drinkers are those who drink only on special occasions, certain months or less than once a week, with options such as “Once a month,” “Less than once a month,” and “2 to 3 days a month.” Regular drinkers are those who drink at least once a week, covering options such as “Daily,” “Twice a day,” “2 to 3 days a week,” “4 to 6 days a week,” “Once a week,” and “More than twice a day.”

### 2.3 Statistical analysis

Descriptive statistical methods were used to summarize the demographic, behavioral, and clinical characteristics of the participants. Continuous variables are expressed as the means ± standard deviations, whereas categorical variables are expressed as percentages (%). To compare baseline characteristics, the chi-square test was used for categorical variables, and *t*-tests were used for continuous variables. All the statistical analyses were performed using R software (version 4.2.2). Two-sided tests were conducted, with a significance level set at *p* < 0.05.

Logistic regression models were used to calculate odds ratios (ORs) and 95% confidence intervals (CIs) to evaluate the associations between obesity and lipid metabolism indicators (continuous and quartiles) and MCR. These models were initially adjusted for age, sex and living area and then further adjusted for education level, marital status, alcohol consumption and smoking status. Given the high convenience and feasibility of measuring BMI, WC, WHtR, and BRI in clinical settings, these four indicators were prioritized for subsequent subgroup analysis, threshold effect analysis, and mediation effect analysis. After adjusting for potential confounding factors, the relationships between obesity-related indicators and MCR were explored through smooth curve fitting and threshold effects. A two-tailed *P*-value of ≤ 0.05 was considered statistically significant.

The “mediation” package in R 4.2.2 was used for mediation analysis to evaluate the mediating role of the CTI in the link between obesity and MCR, adjusting for age, sex, living area, marital status, education, smoking, and drinking. The analysis followed these steps. The mediation model assessed the mediating effect of the CTI between obesity indices and MCR, adjusting for covariates. Significance testing used a bootstrap method (1,000 resamples) to calculate indirect, direct, and total effects, assessing mediator significance (Imai et al., 2010). A mediation effect was defined as present when there was a significant indirect effect, a significant total effect, and a positive mediator proportion.

## 3 Results

### 3.1 Participants characteristics

This study compared the characteristics of individuals with and without MCR. In terms of anthropometric and health-related indicators, the MCR group was significantly older (*p* < 0.001) and had higher CTI (*p* < 0.001), WC (*p* < 0.001), WHTR (*p* < 0.001), CI (*p* < 0.001), ABSI (*p* < 0.001), and BRI (*p* < 0.001) values. In terms of demographic characteristics, the MCR group had a greater proportion of females (*p* = 0.003), a greater proportion of unmarried individuals (*p* = 0.038), a greater proportion of illiterate individuals (*p* < 0.001), and a greater proportion of current nondrinkers (*p* = 0.015). In contrast, there was no significant difference in the distribution of urban and rural areas (*p* = 0.984) or smoking status (*p* = 0.265) (Table 1).

**TABLE 1:** Baseline characteristics of the population stratified by MCR.

MCR	Without MCR	With MCR	*P*-value
**Age**	67.11 ± 6.35	70.86 ± 7.47	< 0.001
**CTI**	8.87 ± 0.87	9.13 ± 0.90	< 0.001
**BMI**	23.51 ± 4.02	24.07 ± 4.03	0.087
**WC**	86.10 ± 10.44	88.95 ± 10.99	< 0.001
**WHTR**	0.55 ± 0.07	0.58 ± 0.08	< 0.001
**CI**	7.47 ± 0.52	7.70 ± 0.48	< 0.001
**ABSI**	8.41 ± 0.56	8.63 ± 0.48	< 0.001
**BRI**	4.42 ± 1.48	5.08 ± 1.75	< 0.001
**Sex**			0.003
Female	49.93%	62.09%	
Male	50.07%	37.91%	
**Living area**			0.984
Urban community	36.52%	36.60%	
Rural village	63.48%	63.40%	
**Marital status**			0.038
Unmarried	19.18%	27.45%	
Married	80.81%	72.55%	
Unknown	0.02%	0.00%	
**Education status**			< 0.001
Illiterate	32.76%	49.02%	
Some primary school/can read or write	21.66%	15.69%	
Finish primary school	24.27%	21.57%	
Junior high or above	21.30%	13.73%	
**Smoking status**			0.265
No	52.30%	56.86%	
Yes	47.70%	43.14%	
**Drinking status**			0.015
Current nondrinkers	66.56%	78.43%	
Regular drinkers	21.39%	12.42%	
Occasional drinkers	11.70%	8.50%	
Unknown	0.34%	0.65%	

### 3.2 Multiple logistics regression analysis

Table 2 indicates that BMI, WC, WHtR, CI, ABSI, and BRI were significantly associated with an elevated risk of MCR. Specifically, in the nonadjusted model, WC, WHtR, CI, ABSI, and BRI showed significant associations. After adjusting for covariates in the Adjust I and Adjust II models, these indicators maintained their significant associations, with slight changes in OR values. Notably, BMI tended toward significance in the non-adjusted model and became significant after covariate adjustment, highlighting the importance of these obesity-related indicators in the context of MCR risk.

**TABLE 2:** Multiple logistic regression equation.

Exposure	OR (95% CI) *P*-value		
	Non-adjusted	Adjust I	Adjust II
BMI	1.031 (0.996, 1.066) 0.084	1.041 (1.008, 1.075) 0.014	1.041 (1.009, 1.075) 0.013
WC	1.026 (1.010, 1.041) < 0.001	1.029 (1.013, 1.045) < 0.001	1.029 (1.014, 1.045) < 0.001
WHtR	309.743 (36.357, 2,638.830) < 0.001	133.648 (13.430, 1,329.984) < 0.001	126.776 (12.947, 1,241.422) < 0.001
CI	2.367 (1.740, 3.221) < 0.001	1.802 (1.308, 2.482) < 0.001	1.805 (1.311, 2.485) < 0.001
ABSI	2.047 (1.551, 2.701) < 0.001	1.483 (1.106, 1.990) 0.009	1.482 (1.105, 1.989) 0.009
BRI	1.286 (1.174, 1.409) < 0.001	1.234 (1.119, 1.362) < 0.001	1.231 (1.116, 1.357) < 0.001

Nonadjusted model adjusted for: None. I adjust the model for age, sex, and living area. The Adjust II model adjusts for age, sex, living area, marital status, education status, smoking status, and drinking status.

#### 3.2.1 Subgroup analysis

There are significant differences in the associations between obesity indicators (WHtR, BMI, WC, BRI) and metabolic syndrome-related risk (MCR) across age and sex subgroups. In the ≥ 65-year-old group, WHtR (OR = 134.67), BMI, WC, and BRI were significantly associated with increased MCR risk, with WHtR posing the highest risk. In males, WHtR had the greatest impact (OR = 530.60) (Table 3).

**TABLE 3:** Subgroup analysis of the associations between obesity indicators and MCR.

Subgroup	OR (95% CI) *P*-value			
	WHtR	BMI	WC	BRI
**Age**				
≥ 65	134.67 (10.37, 1,749.45) < 0.001	1.04 (1.00, 1.08) 0.032	1.03 (1.01, 1.05) < 0.001	1.24 (1.11, 1.38) < 0.001
< 65	116.96 (0.63, 21,610.99) 0.074	1.00 (0.92, 1.09) 0.938	1.02 (0.98, 1.05) 0.317	1.23 (0.97, 1.54) 0.082
**Sex**				
Male	530.60 (8.54, 32,949.49) 0.003	1.07 (1.02, 1.13) 0.006	1.04 (1.01, 1.06) 0.005	1.34 (1.11, 1.62) 0.003
Female	69.09 (4.47, 1,068.77) 0.002	1.02 (0.98, 1.07) 0.256	1.02 (1.01, 1.04) 0.012	1.20 (1.07, 1.34) 0.002

### 3.3 Threshold effect analysis

**FIGURE 2:**
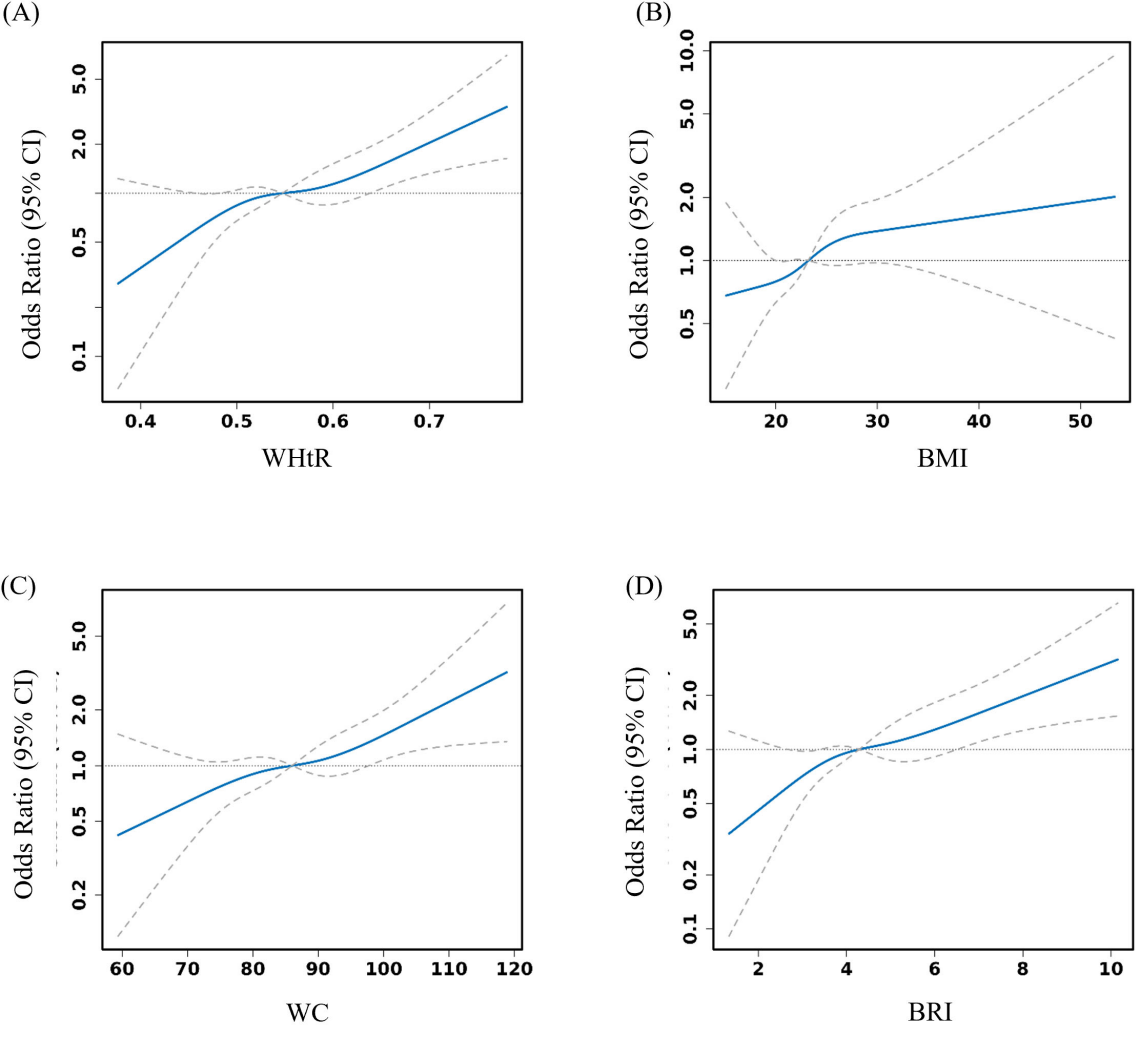
Smooth curve fitting analysis exploring the nonlinear relationship between obesity indicators and motoric cognitive risk syndrome (MCR) (adjusted variables: age, sex, living area, marital status, education status, smoking status, and drinking status). **(A)** It expresses the smooth curve fitting relationship between WHtR and MCR. **(B)** It expresses the smooth curve fitting relationship between BMI and MCR. **(C)** It expresses the smooth curve fitting relationship between WC and MCR. **(D)** It expresses the smooth curve fitting relationship between BRI and MCR.

The results revealed that the potential inflection point of WHtR and WC did not pass the log likelihood ratio test, indicating that there was no significant inflection point and supporting a linear correlation. There is a significant threshold effect between BRI (breakpoint K = 7.059) and BMI (breakpoint K = 29.76) and MCR: below the threshold, the risk of MCR increases significantly with increasing indicators (Table 4 and Figure 2).

**TABLE 4:** Threshold effect analysis.

Exposure	WHTR	BMI	WC	BRI
Outcome: MCR	OR (95% CI) *P*-value	OR (95% CI) *P*-value	OR (95% CI) *P*-value	OR (95% CI) *P*-value
**Model I**				
A straight-line effect	126.78 (12.95, 1,241.42) < 0.001	1.04 (1.01, 1.08) 0.013	1.03 (1.01, 1.04) < 0.001	1.23 (1.12, 1.36) < 0.001
**Model II**				
Folding point (K)	0.45	29.81	70	2.45
> K-segment effect 2	68.30 (6.09, 765.84) < 0.001	0.96 (0.84, 1.09) 0.543	1.03 (1.01, 1.04) 0.002	1.20 (1.08, 1.33) < 0.001
Log likelihood ratio tests	0.069	0.040	0.304	0.041

### 3.4 Analysis of mediating effects

**FIGURE 3:**
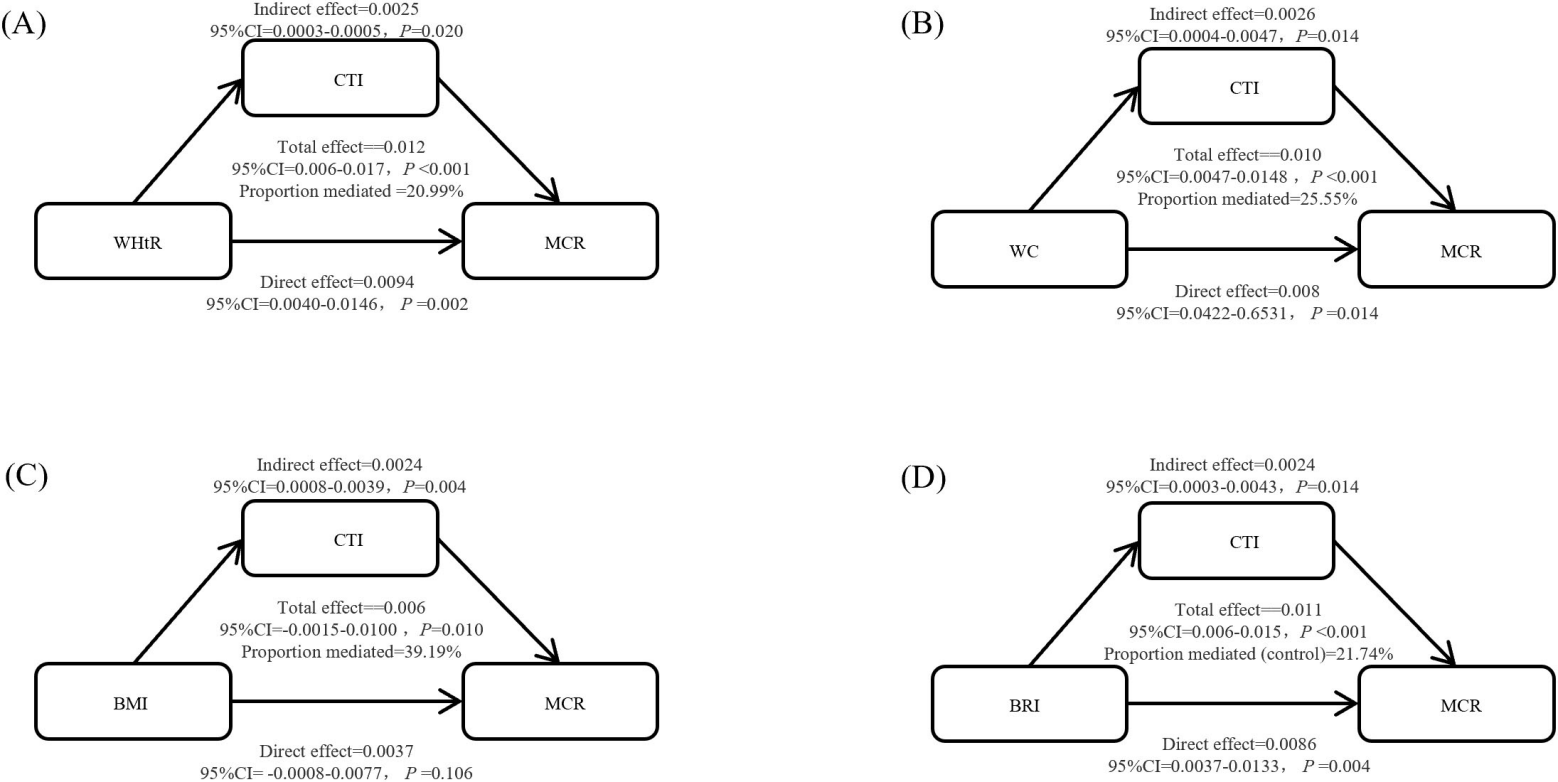
Mediation analysis of the C-reactive protein-triglyceride-glucose index (CTI) in the relationship between obesity indicators and motoric cognitive risk syndrome (MCR) (based on the mediation analysis model, adjusted variables: age, sex, living area, marital status, education status, smoking status, and drinking status). **(A)** It describes the mediating effect of CTI as WHtR to MCR, **(B)** it describes the mediating effect of CTI as WC to MCR, **(C)** it describes the mediating effect of CTI as BMI to MCR, and **(D)** it describes the mediating effect of CTI as BRI to MCR.

Our study revealed that CTI played a mediating role in the associations between WHtR, WC, and BRI and MCR risk, accounting for 16.27, 26.12, and 12.72%, respectively (*P* < 0.05). These findings suggest that the CTI partially mediates the relationship between abdominal obesity indicators and the risk of cognitive decline, highlighting its significance in the underlying pathological mechanisms (Figure 3).

## 4 Discussion

This study focused on the associations between obesity-related indicators and MCR. Through an in-depth analysis of data from the China Health and Retirement Longitudinal Study (CHARLS), several important findings have been made. First, multiple regression analysis revealed that WC, WHtR, and BRI were significantly associated with an increased risk of MCR, whereas BMI was only weakly associated with MCR in some models. These results suggest that central obesity indicators may play a more critical role in the occurrence and development of MCR than overall obesity indicators do, which may be related to the heightened susceptibility of individuals with central obesity to metabolic disorders and neuroinflammatory reactions, thereby affecting cognitive function (Ashwell et al., 2012; Browning et al., 2010; Meiner et al., 2020). Of these, the WHtR has particularly prominent advantages, as it is strongly correlated with both the unadjusted and adjusted models. This may be attributed to its ability to more accurately reflect abdominal fat accumulation, which is closely related to various chronic diseases, such as metabolic syndrome and cardiovascular disease, thereby further increasing the risk of cognitive decline (Ashwell and Hsieh, 2005; Jiang et al., 2023).

This study systematically evaluated the associations between different obesity-related indicators and MCR through a logistic regression model. The results revealed a significant and robust association between the obesity indicators WC, WHtR, and BRI and MCR risk, whereas BMI was only weakly associated. These findings suggest that obesity-related indicators may be important pathophysiological mechanisms for the occurrence of MCR and that the sensitivity of traditional BMI indicators in MCR risk assessment may be limited. First, this study revealed that WC, WHtR, and BRI remained statistically significant in both unadjusted models and after gradually adjusting for confounding factors, with relatively stable effect values (ORs). For example, the OR value of the WHtR remained high at 8.726 in the adjusted II model, indicating a significant independent impact of abdominal obesity on MCR risk. This finding is consistent with previous studies emphasizing the association between visceral fat and cognitive impairment (Isaac et al., 2011; Yoon et al., 2012). Visceral fat can trigger chronic inflammation and insulin resistance by releasing proinflammatory cytokines and dysregulating adipokines, leading to cerebrovascular damage and neurodegeneration (Boccara et al., 2023). BMI may not fully reflect the pathological risk associated with metabolic abnormalities. Notably, the WHtR demonstrated the strongest risk prediction ability in this study. Its OR value is significantly greater than those of WC and BRI, which may be related to its standardization on the basis of height. The WHtR can more accurately reflect the dynamic relationship between abdominal fat volume and body proportion and is especially suitable for risk stratification of individuals at different heights. In addition, as a geometric model indicator based on WC and height, the OR value of BRI (adjusted for Model II OR = 1.041) is lower than that of WHtR but still significantly higher than that of BMI, indicating its potential clinical value as a novel abdominal obesity indicator. These results are consistent with recent studies calling for the inclusion of abdominal obesity indicators in cognitive impairment screening (Chen H. et al., 2024; Hu et al., 2024). This study has certain limitations. First, the cross-sectional design makes it difficult to clarify the causal relationship between obesity indicators and MCR, and prospective cohort studies are needed to verify the temporal association. Second, despite adjusting for multiple confounding factors, residual confounding factors such as dietary patterns and genetic susceptibility may still affect the results. In addition, the racial and regional characteristics of the study population may limit the generalizability of the conclusions, and further validation is needed in different populations.

The results of the threshold effect analysis indicate that there is no significant threshold effect between WHtR and MCR; that is, there is no clear WHtR value. Below this value, the risk of MCR changes sharply, which may indicate that the impact of WHtR on MCR risk is more inclined toward a linear pattern overall or that its potential nonlinear relationship is more complex and cannot be accurately identified through current samples and methods. The threshold effects of the BRI and BMI indicate that within a certain range, the risk of MCR increases with increasing values of these two indicators. However, when a specific threshold is reached, this association significantly weakens, which may reflect differences in the susceptibility of the human body to metabolic disorders at different fat contents. In contrast, WC only showed a significant increase in risk in the high-value range, but the threshold effect did not reach statistical significance. This may be related to the limited predictive ability of WC as a single abdominal fat indicator for MCR risk in different population characteristics. Overall, the relationships between different obesity indicators and MCR present diverse threshold effect patterns. The linear correlation of the WHtR suggests that it may be more suitable for assessing overall trends in the assessment of MCR risk, whereas the threshold effects of BRI and BMI help identify turning points in MCR risk at specific body fat levels. This is highly important for precise clinical intervention and the formulation of public health strategies.

The results of this study indicate that BMI is a risk factor for MCR in the male population but is not significant in females. The reason may be that men are more prone to visceral fat accumulation, whereas women tend to have a greater subcutaneous fat distribution. Previous studies have reported that a nonlinear association between BMI and type 2 diabetes, glucose, high-density lipoprotein cholesterol, and triglycerides has been found only in men (Mutie et al., 2023). Visceral fat is more closely related to insulin resistance and chronic inflammation, and these mechanisms can increase the risk of MCR by affecting cerebral vascular function or neuroinflammatory pathways (Xie et al., 2022). In this study, BMI reached statistical significance (*p* < 0.05), but the effect size was small. Given the small observed effect size, caution should be exercised when these findings are interpreted.

The sex-based difference in the link between WHtR and MCR results from biological and behavioral factors. On the biological side, premenopausal women have greater insulin sensitivity than men do, likely due to the protective effect of estrogen; however, obesity can weaken this effect, and men show worse insulin secretion dysfunction at the same obesity level, so a WHtR increase is associated with greater metabolic risk for men (Gado et al., 2024). Behaviorally, men are more prone to high–risk behaviors, have lower exercise compliance, poorer dietary patterns and weaker health awareness (Kholmatova et al., 2022), which along with abdominal obesity heightens metabolic risks. Future research ought to take sex differences into account.

The mediation effect analysis further revealed that CTI plays a significant mediating role in the impact of WHtR on MCR, mediating 20.99% of the effect, and that the total effect, direct effect, and indirect effect are statistically significant. In addition, CTI also had significant mediating effects on WC and the BRI, mediating 25.55% and 21.74% of the effects, respectively, with direct effects dominating both. As a comprehensive indicator, the CTI reflects inflammation, metabolism, and blood glucose levels in the body. The role of CTI in mediating the effect of WHtR on MCR suggests that CTI may affect MCR by influencing the body’s metabolism and inflammatory status. The CTI integrates inflammatory markers (CRPs), lipid metabolism indicators (TGs), and glucose metabolism indicators (glucose), and therefore reflects the chronic low-grade inflammatory state caused by visceral fat expansion (Islam et al., 2024). Visceral fat expansion triggers inflammation, which in turn exacerbates metabolic disorders such as insulin resistance and hyperglycemia. CTI can mediate 12.7%–26.1% of obesity-related metabolic cognitive risk (MCR), and its mechanism may be related to inflammation-mediated vascular insulin resistance. In terms of clinical application, the CTI can be used for community screening of individuals who may be at risk for central obesity. This approach requires only routine blood tests, which are cost-effective. In terms of intervention, measures such as the Mediterranean diet and GLP-1 receptor agonists can modulate the components of the CTI (Thornton et al., 2024).

The WHtR and BRI, which are indicators of abdominal obesity, are significantly correlated with MCR and exhibit a nonlinear threshold effect. Chronic inflammation caused by abdominal obesity is an early trigger for insulin resistance and cognitive impairment (Abi Saleh et al., 2022). Inflammation-mediated vascular insulin resistance can occur early in obesity, further exacerbating metabolic disorders. Proinflammatory cytokines secreted by adipose tissue, such as interleukin-6 and tumor necrosis factor-α, can induce neuroinflammation by damaging the blood–brain barrier (Mruczyk et al., 2025), which is in line with the mediating effects of C-reactive protein, triglycerides, and the glucose index (CTI) in this study. Future research should incorporate data from multiple time points to analyze the associations between the rates of change in obesity indicators and the progression of MCR.

With respect to interventions, increasing evidence suggests that glucagon-like peptide-1 receptor agonists (GLP-1RAs) may become a precise treatment strategy for MCR. GLP-1RAs can reduce visceral fat and inhibit the infiltration of adipose tissue macrophages (ATMs), thereby directly alleviating chronic low-grade inflammation associated with obesity (Hropot et al., 2023). Relevant clinical studies have shown that the use of GLP-1RAs by obese individuals significantly reduces the risk of Alzheimer’s disease and vascular dementia (Siddeeque et al., 2024). Animal experiments have indicated that GLP-1RAs can protect synaptic plasticity by inhibiting the abnormal activation of microglia, which is closely related to the pathophysiology of the motor–cognitive dual impairments in MCR (Henn et al., 2023). The WHtR association results and CTI mediating effect in this study suggest that GLP-1RAs may be most effective for individuals with abdominal obesity and metabolic abnormalities.

However, this study also has certain limitations. Despite the use of a longitudinal study design, certain challenges remain in fully determining causal relationships, as there may be confounding factors that have not been measured or adequately controlled for affecting the results. In addition, the research sample was sourced from CHARLS. Although it has national representativeness, its applicability in certain specific populations or regions may be limited. In the future, the sample size can be further expanded, or multicenter studies can be conducted to increase the generalizability of the results. Moreover, there may be certain errors in the measurement of some indicators, such as measurement accuracy during physical examinations and recall bias during questionnaire surveys. Subsequent research can explore more accurate measurement methods to reduce errors.

## 5 Conclusion

This study is based on data from the China Health and Retirement Longitudinal Study. Through systematic analysis, the following conclusions were drawn: waist circumference (WC), waist-to-height ratio (WHtR), and obesity index (BRI) are significantly positively correlated with MCR risk, with WHtR having the strongest predictive power (adjusted OR = 8.64), and its effect follows a linear pattern (*P* threshold effect = 0.159), suggesting that central obesity indicators should be prioritized over traditional BMI in clinical screening. CTI, as a complex biomarker integrating inflammation and metabolism, accounted for 22.2% of the WHtR MCR associations, confirming that metabolic disorders constitute the core pathway through which obesity affects cognitive function. Subgroup analysis further revealed that risk heterogeneity (WC, WHTR, and BRI) was more strongly associated with MCR than BMI was, particularly in older adults, women, rural populations, less-educated individuals, and alcohol consumers. This study innovatively discovered the threshold effect of the WHtR and BRI: when the BRI < 7.06 or BMI < 29.90, the risk of MCR significantly increased with increasing indicators (OR = 1.23 and 1.08), but the effect weakened after exceeding the threshold, providing a quantitative basis for precise weight management. Although the cross-sectional design limits causal inference, a pathological pathway framework of “central obesity metabolic inflammation MCR” was constructed through multiple model adjustments and mediation analysis. In the future, prospective cohort validation of temporal correlations is needed, and multi-omics techniques should be combined to analyze the molecular interaction mechanisms among fat distribution, metabolic factors, and neurodegeneration. These results provide evidence-based support for early screening of cognitive impairment in elderly individuals and suggest that the WHtR and CTI should be incorporated into the community health assessment system to provide a scientific framework for efficient prevention and control in resource-limited areas.

## Data Availability

The original contributions presented in this study are included in this article/Supplementary material, further inquiries can be directed to the corresponding author.
